# Report on the Influence of Civilization in Lessening the Number and Severity of Diseases

**Published:** 1844-07

**Authors:** K. F. H. Marx

**Affiliations:** Professor in the University of Göttingen


### REPORT ON THE INFLUENCE OF CIVILIZATION IN LESSENING THE NUMBER AND SEVERITY OF DISEASES.

By K. F. H. Marx, M.D.,
Professor in the University of Göttingen.*

It is a complaint often repeated, that our present epoch, although advancing in an economical and even in a mental point of view, is nevertheless retrograding in regard to the physical condition of man. It is said that the present race of mankind is feeble when compared with that of former times, and that diseases have increased, as well in their number as intensity. Many reasons seem to favour this opinion, especially the over-refinement of our social habits and enjoyments; the enlarged nomenclature of diseases, and the increased number of institutions for their cure.

This conclusion, however, although it may seem convincing at first sight, will appear utterly groundless if properly tested. It will therefore be our task to show, that, as civilization extends and increases, the sanitary condition of nations and individuals improves in the same ratio; that diseases are really on the decline, as well in number as intensity; and that every advancement in the path of knowledge and morality exercises a salutary reaction on the whole bodily condition of the human kind.

By means of advancing civilization, population not only goes on increasing, but even the duration of individual life becomes longer, and life itself less subject to the encroachment of disease. Plagues, which were considered by antiquity, and even more recently, as necessary evils and as trials of mankind, are now known only by name; and others, which were counted amongst the necessary consequences of human presumption, and as natural barriers to a state of happy existence on earth, are now easily accounted for and remedied. Several maladies which used to decimate mankind and destroy the organs of the senses and the beauty of the human form, are nearly extinguished. Others, which were considered as indispensable consequences of the progress of trades and professions, or even of the mere reunion of men in towns and cities, a more refined knowledge of hygiene has shown to be only adventitious intruders, and, as such, has successfully warred against them.

At first sight, we may readily believe that a free condition of life—one, as it is called, in accordance with nature—is the most advantageous to man, and that we ought to devote ourselves to agriculture, the pursuits of the huntsman or fisher-

* This Report is taken from the second volume of the *Transactions of the Royal Society of Sciences of* Göttingen, published in 1844; it was read before the Society in May, 1843. The present translation having been made, in the first instance, by a German imperfectly acquainted with the idioms of the English language, it was found necessary to submit it to a complete revision; and although considerable pains have been taken to render the style more idiomatic, it will still be found *foreign* in many respects.

It has been thought proper to give the Report, as it stands in the original, without note or comment; but the intelligent English reader will not fail to remark more than one passage where the enthusiasm of the learned and amiable author has led him to employ his rose-colours rather too freely. Civilization has done much, doubtless; but it has not done all that the kind-hearted Professor hopes and believes.—Ed.

man, if we wish to enjoy a degree of uninterrupted well-being. Every walk which we take in the open air, every sojourn in the country, every journey,—all of which so quickly and wonderfully refresh and soothe both mind and body,—seems to prove that the intimate intercourse with nature conceals the secret of health; and that every deviation from her paths is accompanied by disease and imperfection. Although this may be true, to a certain extent, we must not lose sight of the difference which exists between resorting to nature for a limited period and for recreation, and the constant following of rural pursuits as a means of subsistence. The peasant, the fisherman, the huntsman, can tell us of other concomitants and consequences of their respective callings, besides those of mere pleasure and enjoyment. The greater part of what nature yields must be extorted from her by exhausting toil, or obtained by a scarcely less exhausting patience. If the higher faculties of man are not properly excited and used, the whole mind will fall into a state of stupor and "mere oblivion." Perfect health is not possible without an adequate harmony of the bodily and mental faculties. An individual who possesses nothing but mere bodily health approaches very closely to the mere animal. Each individual man, as well as the whole race, has assuredly a very different vocation and one much more exalted than that of merely preserving health. And it will be our business to show that the tendency of civilization is to invigorate the physical man, while it promotes other endowments and creates new ones. The development of the higher faculties does not infer the manifestation of the dreaded evils. Only that mental development will be noxious which is effected without due consideration of the time requisite for its production, or of its legitimate amount, and the proper means of acquiring it. True civilization knows best how to keep within adequate bounds, and to follow that line of conduct which answers all reasonable demands.

It is true, that the many senseless claims of society, the arbitrary dictates of conventionality and fashion, and other agencies called forth by the passions and by party strife, will often cause an interruption of health, either constant or transitory; but it is equally true that by means of the prudence, firmness, and calculated action which are the result of genuine civilization, the deleterious effects of those agencies will be either avoided or much lessened. At any rate, we cannot believe that these dark spots of civilization are sufficient to obscure the value of its brighter parts. The mental activity consequent on social cooperation, the numberless impulses which, engendered by objects of art, strike and vivify our senses, the endless impressions produced by reading, instruction, example, &c. excite, in a most important degree, the bodily functions also, and impart to them new power and energy, with a higher development of the intellectual faculties, an augmented strength of moral character, and increase of religious resignation: man becomes more capable of resisting the injurious influences of external nature; knowledge and a certain moral equipoise are found to be the most efficacious means for acquiring that pliancy and elasticity which enable us best to support the severest bodily pains. Accordingly, we believe that the robust pupil of untutored nature, deficient as he will be, in the case of severe illness, of every assistance of a higher character, will, speaking generally, be found to succumb much sooner to the same diseases than the delicate and even weakly offspring of civilization, to whom every advancement of science and art, the intellectual treasure of books, society, and conversation, will constantly minister fresh sources of life.

The reports of travellers who have sojourned for a long time amongst savage tribes are very discrepant, as far as their remarks on the sanitary condition of the people are concerned. While some speak only of a few maladies there observed, others acknowledge to have met with most of our principal ailments. But suppose even that a traveller has observed but few maladies in any place, it does not follow that they occur but rarely there. May the cause not rather lie in the inhumanity of the inhabitants, fostered by want and sanctioned by custom, and in the insufficiency of medical treatment? Those very diseases which most obtrude themselves on observation—those of a chronical or lingering character, which destroy the body by degrees, and against which art, with us, makes a determined stand—only yielding to them step by step, and even often succeeding in vanquishing them altogether—such diseases are scarcely to be met with among savages, because at the very first appearance of any such, the patient is carried off, either through neglect or want of proper management.

It is well known that fractures of the legs of animals seldom heal perfectly—a circumstance which is to be accounted for by the want of proper quiescence of the part: the animal will not lie down. The owners will not risk the trouble and expense of the perfect cure, (at best only problematical;) and the animal is put out of the way. Can this be adduced as a proof that fractures of bones are of rare occurrence among animals? Nations and tribes who have to defend themselves against enemies, and constantly to busy themselves about the procuring of their subsistence cannot pay the necessary attention to persons affected with chronic diseases. Mere kindness is soon exhausted; the impulse of self-preservation overcomes, in the long run, even the natural sentiments of near consanguinity, and a stupified indifference leaves the victim to its fate.

In a community where every one needing sustenance must necessarily seek for food, or earn food, those alienated in mind, who are unable to share in any work, but rather impede every useful undertaking, cannot be much taken care of. Lunatics are like persons in a state of trance, whose revivification can only result through a continuous personal self-denial. If such persons are left to their fate, under all sorts of privations, they must soon succumb; they cannot, therefore, so often meet our eye as with us, where everything is done for their preservation and comfort. We shall hardly, therefore, be tempted to regard the life of any nation or tribe, which approaches the so-called state of nature, as an object of envy to civilized man.

But, in order to place the true influence of civilization on the physical weal of man in the proper light, it is not enough to prove that the increase of disease caused by it and through it, is in some degree only apparent; we must also concede at once, that there is a great number of morbid causes contingent on civilization, which, although they are necessarily connected with it, still are capable of being mitigated, neutralized, or removed by it: as in the Grecian fable of the spear, the same thing both kills and cures. One of these causes—although one perhaps not yet fully recognized—is the remarkable fact, that while the populousness of most countries goes on continually increasing, the mortality of individuals, on the other hand, is continually decreasing. The longer lives of the present generation, compared with those who preceded it, and the diminished risk from the diseases of infancy and youth, have left more room for disease in the enlarged subsequent period of life.

A consequence, however, of increased civilization, scarcely to be avoided, is, that through the improvement of mechanical art, and the greater number of manufactories, the mass of working men who possess no fixed property (land), is also on the increase, by which the contrast between comfortable ease and hopeless misery becomes the more apparent. Is it not to be supposed that the pauperism of the working classes—which St. Simonists, Fourrierists, Socialists, and Communists have sought in vain to remedy—is a never-failing source of disease? So is it not to be supposed as well, that the use of ardent spirits, (once counted amongst drugs, and not used as a popular beverage before the beginning of the sixteenth century,) but now so much misapplied, may be alone sufficient to account for the greater number of sick, compared with former times? Although both these queries must be answered in the affirmative, yet it can be also proved, that along with these unavoidable drawbacks of a highly-developed civilization, the means to obviate them and to confine them in a narrower channel, also become manifest and multiplied.

One source of agencies leading to disease seems to lie in the very development of the human mind. The more varied and the more powerfully the mind is brought into action, the more numerous will be the occasions for the morbid misturning [disorder] of the higher parts of the organism. A proof of this may be found in the fact, that the lunatic asylums are everywhere either increased in number or enlarged. This seems to accord with a saying of the old Stagyrite, that men distinguished by talent, either as philosophers, statesmen, or poets, or in other arts, are prone to melancholy; which is corroborated by the statement of a recent Belgian political economist, that it is between the age of forty and fifty, when most of the great works of the human understanding are accomplished, that man is also most subject to mental alienation.

A more accurate investigation into the circumstances of the case, however, will teach us how uncertain and onesided such assertions are, leading to erroneous conclusions. Even the very logical meaning of words is here of great importance. When the ancients called a person melancholy, they did not mean, any more than we do, that the individual was diseased; but wished to express merely that stern retirement within one's self which men of genius feel at times, as if they were compelled by a higher and irresistible agency. Genius, the superior development of mind in any given direction, will scarcely ever of itself lead to disease; and if this should ever chance to be the case, it will be so often reported that it may grow at last into seeming importance by repetition. Men who outstrip their contemporaries by their actions or by other mental manifestations, are considered eccentric and even mad. It would be well if, in such cases, we could interrogate history as to particulars. If we hear that the poet of 'Gerusalemme Liberata' was a lunatic, it would be well to inquire whether this was really the case, and if so, how he was brought into this state. Richly-gifted minds, as well as those most obtuse, may become affected by psychical disease; but with the former it is considered a phenomenon, with the latter an every day's occurrence. It is certainly a very hasty conclusion, that the development of all or any individual mental quality can be the cause of mental disturbance or even disorganization. Not culture, but half-culture of the faculties leads to the lunatic asylum. The more numerous and the better the educational institutions of a country, the less the number of lunatics. The more all capabilities of man are brought into action, the more perfectly will all imperfections be overcome. The want of activity will much oftener cause mental disturbance than activity ever can. How seldom is it that men really learned, who work quietly and in measure, are mentally diseased! It is not the exertion of the mental faculties and the strenuous efforts for the achievement of the highest aims of existence which will disorder the higher mental powers; but rather passions and the accidents of fortune, against which elevation of the mind is the surest remedy. If, therefore, it has been stated by high authority, that the progress of so-called civilization increases the number of suicides, it is only the spurious, not the genuine civilization which is to be blamed. The latter will even teach us that the aim of life does not consist merely in earthly enjoyment, and that it is our bounden duty to bear heavy visitations with resignation.

Whether the number of lunatics is really on the increase, compared with former times, on account of greater civilization, cannot be ascertained, as no, or at least very incomplete, numerical accounts were kept at former periods. Even the most recent reports of lunatic asylums are not entirely to be depended upon. As little as the court fools of the middle ages were real fools, as little do all actually-reported lunatics belong to that category. Oxford, who discharged pistols at the queen, and is now confined in Bedlam, is certainly not a lunatic. Formerly such unfortunate persons were kept secluded in private houses, partly for the sake of keeping such family afflictions secret from the public gaze, partly in order to avoid any extraneous interference. Now they are generally sent to institutions of that kind. Asylums formerly were employed for safe keeping, now for cure. Insane persons, in past times, were only attended to at the end of their malady, or on the occurrence of some imminent danger from them; now proper treatment is applied at the very outset of the malady. For all these reasons, more spacious and additional buildings have become necessary for the accommodation of lunatics.

So far, therefore, from civilization being justly considered as a nursery of mental disease, it stands forth, as far as their cure is concerned, as the advocate and promoter of the purest humanity. Never has sympathy or the devoting forgetfulness of self burst forth into finer bloom than in many of the present institutions for mental alienation. The further we advance in the knowledge of maladies of this sort, the more forms thereof we become acquainted with. But it does not follow thence that such did not previously exist. On the contrary, some varieties of mental disease which existed in former times have already disappeared, or are on the point of disappearing. One species of lunacy, viz. *lykantrophy*, has really ceased. In the third and fourth centuries maniacs were seen prowling and howling about on graves, and other solitary places, like wolves; and they were rather numerous in several parts of Europe.

That form of innate imbecility called cretinism, which seemed hitherto to withstand any attempt of cure, has been alleviated and even cured now-a-days, by a successful combination of medical art and philanthropic expedients.

Animals were once called "the mutes of nature," out of compassion; the term implying that the organ of communicativeness was denied to them. It is only at a recent period that notice has been taken of those most unfortunate people to whom nature has denied the use of hearing, and thence prevented the use and development of their organs of speech. Philanthropists and instructors and medical men have united in promoting inquiries into the causes of dumbness and deafness, according to certain localities, and as well into their varieties, and have exerted themselves in making the lot of such persons as comfortable as possible. Formerly the deaf and dumb were a great burden on human society. Not taking into account those few who, by otherwise favorable circumstances, were permitted to attain some degree of moral and social independence, the remainder, if left to their own helplessness or the brutality or stupidity of those who commonly surrounded them, became objects, not only of compassion, but even of terror. How differently stand things now with this class of persons, brought up and educated as they are in public institutions! Here, by being instructed in reading and writing, the power of comprehending and mingling almost on equal terms with the remainder of society is opened to them; a substitute being supplied to the hitherto closed up and unyielding organs of speech and hearing! Be the causes of this abnormal state what they will, it is certain, that civilization has no share in their production, while it is from it alone that their only possible alleviation is proceeding.

The same may be said of all institutions for the reception of the blind, deformed, crippled, lame, &c., whom an increased knowledge and experience teach us to heal or to relieve. Evils of this kind have always existed, but in past times they were only productive of pain and care, both to the individual and to society at large.

In the same way as the *cure* of bodily ailments is attended to, so also pains are taken for their systematic *prevention;* and it is really not the fault of civilization, if the primordial germs of malady are continually sending forth fresh branches.

It would be easy to corroborate the foregoing assertions, *seriatim*, but this examination would lead us here too far. But the enumeration of some of the most important efforts against the spreading of maladies—efforts owing to the advanced state of civilization—will convince us that this decrease is not owing to any accidental epidemic influences, or to periodical cycles, but solely to the strenuous efforts of the human mind. It will show, at the same time, the constant manifestation of efforts either to limit the causes of maladies, or to eradicate them altogether, their progress being opposed at every step.

In turning our regards backwards, we shall find that each succeeding age, every advance in civilization, is marked by increased attention to the physical condition of children, and, consequently, by their decreased mortality. This attention begins even before they are born. How much more do we now (compared with former times) pay attention to the difference between the natural and artificial indications during the act of parturition, and thus by a well-calculated, timely aid, save the life both of mother and child. As the case may require, a healthy wet-nurse is procured, or the child brought up, at least, in an appropriate manner.

Much is done towards the preventing infanticide, whether intentional or that which may be caused by the want of proper knowledge. Orphans, or otherwise friendless children, are sent to the country to be nursed, not only for the purpose of avoiding their being placed in foundling-houses, but also for the reason, that in the first period of life the mortality of children is greater in towns than in the country.

Education tends now as much towards the developing of the physical as of the mental faculties; and if there be any hereditary disease, care is taken to check the elements of its growth by an adequate mode of life.

Our mode of dressing is more appropriate than formerly, and such garments which prevent, by their pressure, the free action of the organs, fall every day more into disuse. The less common use, or the better construction of stays, has done away with many a complaint. The same observation applies to our cropped and cleanly-kept hair. Dentition is scarcely ever mentioned amongst the diseases of infants, since the too warm keeping of the head has been abandoned.

The conviction of the necessity of dietetic and gymnastic invigoration of the body, as well for youth as those more advanced in age, is gaining ground every day. The means for accomplishing this desirable end, which heretofore was only within the reach of some classes of society, or was peculiar to certain races of men, are well-nigh becoming the common property of all nations.

Considering the important influence which the skin exercises on the most important functions of the body, the habits of cleanliness, which have now become a custom, contribute powerfully towards the preservation of health. It has been much aided by the universal use of soap. It is with perfect truth that cleanliness is considered one of the most valuable gifts of civilization.

With the increased knowledge of the conditions requisite for general welfare, the endeavours for maintaining these, and carrying them out, have kept equal pace. That weighty saying, "what maintains health, maintains wealth," is daily corroborated by manifold experience. Almost everywhere great efforts are made to keep the air pure and sweet, by the laying out of broad streets, forming sewers, and the removal of cemeteries from the vicinity of the habitations of men. Since the general peace of nations is now firmly established throughout Europe, high bastions may well cede their place to a free access of air, and narrow confining walls, and malarious ditches, be changed into public walks.

The supply of healthy food is so much facilitated, that diseases which formerly spread over vast tracts of land, on account of its deficiency, are now nearly unheard of. The scientific improvement of agriculture has made all sorts of soil available, and the amount of field produce has increased. The abundance of potatoes and fruit alone is sufficient to prevent scarcity, or real want.

The adulterations of nutritious substances, the glazing of culinary vessels in a manner deleterious to health, the imperfect tinning of kitchen or other economical utensils, are becoming every day more rare. Cases of accidental poisoning decrease every day in number, by the progressive destruction of venomous herbs, or by their character becoming better known. The sale of poisons is subjected to rigorous control, and the vending of patent medicines (*Olitäten krämer*) entirely prohibited [in some countries]. If a case of poisoning occurs, efficacious antidotes, the result of experiment and research, are at hand. From the high degree of development which chemical art has attained, the ascertaining of any poison introduced into the body is a task so sure and easy, that all premeditated poisoning is easily ascertained, and its repetition made more difficult.

Active and efficient relief of the poor is carried everywhere into effect. The distributing of warm clothes and fuel in the winter season, the numerous societies for providing convalescents with adequate food, the institutions for destitute infant children—all branches of the one charitable feeling towards the poor—contribute powerfully towards the preservation of life and health in the lower classes. The establishment of poor-colonies (home-colonies), tried lately in several countries, bids fair for a permanent improvement of our social state, partly by relieving the community from an onerous surplus of population, partly by the increased happiness of thousands of needy people, and partly by bringing into cultivation tracts of land, some of which would otherwise remain a constant source of deleterious effluvia.

Those arrangements whereby prisons become not merely places of punishment, but of reformation, are constantly more and more extending themselves; consequently a great number of people are not only physically but morally preserved. The merits or demerits of the several methods of treating prisoners are by no means yet perfectly ascertained. Still, the time is not distant, when those philanthropists interested in this weighty object, will agree on some final plan proved to be the best. If the fact, that the American penitentiary system is often followed by aberration of mind should become confirmed, it will, of course, prevent its adoption.

The generally milder character of punishments of the present age, cannot fail to preserve the health of those who have subjected themselves to such treatment. It is only from tradition, as it were, that we know in civilized states of organic defects or mutilations having followed brutal punishments.

In the military service, also, a humane treatment is gradually superseding the old strenuous severity. It is not merely attention to cleanliness and sound food, but also a humane treatment, which can prevent disease of the common soldier; so much so, that the saying is not far from truth, that "a good commanding officer has generally a healthy troop." To the establishment and the improvement of military and field hospitals, thousands owe the preservation of life and limb.

Since care has been taken that not too many persons are crowded on board of ship, that the crew are provided with a sufficient supply of linen, and that instead of the old air-infecting ballast, blocks or rather tanks of iron (containing the supply of water) are had recourse to, the salubrity of vessels has made astonishing progress.

The special investigation of the diseases of artists and artisans has greatly tended to ascertain the hidden sources of their ailments, as well as the ways and means for removing them. How many owe to the drawing-oven of D'Arcet, or the safety-lamp of Humphry Davy, their preservation from lingering disease, or even death! Many operations which formerly, from the unnatural position and exhausting toil required in them, destroyed the health of the workman, are now accomplished by means of machinery.

The fact that the mortality is greater in manufacturing countries and districts, than with people who live in the country, seems to be partly based on the circumstance that the sources of employment do not flow steadily in the former, and that not seldom the flood-tide of enjoyment is followed by the protracted ebb of privation. The beneficial effects of savings'-banks, every day more and more admitted, will eventually succeed in obviating this inconvenience.

Even the nursing of the sick in hospitals and private dwellings, inasmuch as this now demands as an indispensable requirement, the proper separation of the patients, free circulation of air and cleanliness, has thereby elevated itself into an important branch of the sanitary art. Equally beneficial in that respect, as well as productive of general good, and indispensable for obtaining scientific results, was the establishment of separate institutions for sick children, for incurables, for those affected with skin diseases (*Krätzige*), syphilis, epilepsy, &c., together with separate wards for convalescents, especially those labouring under contagious and mental diseases.

The preservation of those who had received severe injuries, as well as the asphixiated, did not remain dependent merely on the sympathising feelings and the skill of individuals; public solicitude exerted itself in this behalf. Prizes were given to those who had saved the life of others, as well as to those composing the best essays on the means of doing so; instructions also for treating such cases, intelligible to common persons, were distributed, and societies were formed for their relief, which took the exclusive appellation of "humane."

Contagious diseases are repressed in the same proportion as the authorities carry out preventive measures: all suspected articles undergo several processes of purification, such as exposure to air, washing, calefaction, and often they are even burnt altogether. The discovery of the preparations of chlorine has afforded a strong means for checking putrefaction and infection.

A more careful study of veterinary medicine has taught us what animal contagions can be communicated to man: and the knowledge of these facts points out the necessary care, and the means which secure man against them. The often repeated muster of dogs, the speedy removal of all which are suspected, and the taxes which have been laid upon them, with the view of leading towards their decrease,—all these expedients have been so efficacious, that for several years past no case of real hydrophobia has come under our observation.

The eradication of endemic agencies has been much advanced by medical topography. It points out the influence which the soil and climate in general exercise on the inhabitants of various localities. Wherever man penetrates with his peaceful arts and occupations, and his free institutions, bogs, dense forests, and with them the concomitant cold and damp, will vanish. But in the same ratio also in which the commercial and political importance of a country are declining, and industry and population diminished, the malaria, and especially the marsh-miasma, will increase.

The great share which scientific discussions on sanitary subjects has had in the preservation of health is not to be passed over in silence. In the same category comes the publication of good popular works, now issuing from the press in several countries of Europe, especially in England, where they are composed by first-rate men, and sold at most moderate prices. The opposition to prejudices of every kind, be it by oral discourse or by the public prints, gains every day more ground. The more we find that public health is encroached upon by prejudices and abuses, which are based either in ignorance or selfishness, the more unrelaxed ought to be our opposition. As long as the opinion prevailed that the burial in churches and chapels, under the immediate protection of saints and martyrs, led to salvation, the faithful were doomed to inhale the vapours of graves.

That the burning of widows is no necessary proof of affection has been duly acknowledged by the English in India, and they have prohibited it, as well for reasons of humanity as of sound sense.

There were times when either all or some diseases were considered as punishments or visitations sent by God, to which man had to submit without resistance. These false notions have, however, long passed away; or if such a dogma of predestination is still in force in some countries, it is to be hoped that it will soon vanish before the light of reason. As in this case from *religious* scruples, so in the case of vaccination arguments founded on a basis of *humanity* were advanced against that most beneficial measure: it was then considered improper to convey to the human body an animal virus, and thus to exchange the dangerous human varioli for the innocuous form of the cow virus. Nowadays such things are smiled at.

From a misunderstood delicacy in difficult deliveries, the right moment for relieving both mother and child was formerly neglected; but now, since the repugnance of the female sex against male interference has been overcome, and proper aid being now called in at the right period, many a woman and her offspring are saved.

Many habits and customs, connected with sanitary laws, do not assume their legitimate and natural bearing but by slow degrees. The practice of keeping the patient warm, especially in febrile exanthematic diseases, in scarlatina, and miliary fevers, and even in measles, was pushed to such an extreme, that it became a true calamity. This air-repugnance, if we may so term it, is now vanquished; nurseries and sick rooms are ventilated; no one now forbears to carry children into the fresh air, to wash invalids with cold water, or even to pour it over them; and if the new system of hydropathy has carried its precepts too far, still even this lapse into extremes is on that side where danger is least, and the return into the right path nearest at hand.

As the spread of genuine civilization is capable of stopping the increase of disease, so is it also with the advance of morality. The shutting up, for instance, of gambling houses, stops also a considerable source of morbific agencies.

Philanthropic societies accomplish, by their wide-spread activity, not only the high aims of civil and moral improvement, but also increase health and prolong life. Who ever can mistake the blissful effects of temperance societies, even conceding that their activity is much crippled in many countries? The command which the man of cultivated mind can exercise over himself, by the means of principle and firm will, must be impressed upon coarser minds by example, at times even by a sort of monastic vow. Temperance, however, is the chief basis of all physical prosperity, the foundation and preservative of every human blessing. If modern time can succeed in erecting everywhere temples to that divinity, it will have fulfilled a high vocation. The reason why more *men* die in towns is, because their life is, in the main, more disorderly.

Life-insurance companies, and all such arrangements whereby that which we have acquired or saved can be deposited for the time of want, advance, in so far, the physical welfare, as they protect us against the barassing and corroding vicissitudes of life.

If the fact be true, that the prosperity of the lower classes is increasing, it will not fail to exercise its influence also on their general physical well-being. Many a child of the poor, which looks well at first, dwindles away gradually, verifying the opinion, that in proportion as misery decreases, more infants are reared up to adult life. The more roomy dwellings, better protected against cold and wet, as well as better clothing and food, will keep off many a bodily ailment, especially where infection is to be apprehended. How many diseases are to be met with in the hovels of misery and privation! Blindness is most common with the poorest classes, as it chiefly originates in neglect and want of proper management.

The habit of being much in the open air, and the consequent hardening of the body, contributes much towards the preservation and prolongation of life; but if this condition be accompanied with severe labour, it will lose a great deal of its benign influence. Hence it comes, that the so-called simple states of life do not prove themselves so beneficial in this respect, as those of more civilized nations. In like manner, we know, that more women die during the time of their pregnancy in the country than in the towns. It may be, that this is at times caused by the want of proper help, but still more we think it is owing to the necessity women are there under of doing hard work at a period which demands great quiet. Considered, however, generally, we know there is less mortality in the country than in large towns, and that some sorts of maladies are rarer there; but the cause of the difference is not to be sought for so much in the production of noxious airs, consequent on the concentration of so many people, but rather in the fact that all agencies tending to the disturbance of health and life are there more copious. The more, however, these agencies are acknowledged as such, the greater will be the care of all well-informed men, and of philanthropists and the public authorities, to diminish them.

The mortality amongst the higher classes, compared with the lower, is not only less, because the former live in plenty, and the others in want, but also because the former are accustomed to cleanliness and temperance, are less excited by low passions, and less subject to sudden changes of their circumstances. It is remarkable that in England, where no doubt there is the greatest amount of general material well-being, the average duration of human life is also the greatest, viz. thirty-eight years, whilst in Russia it is only twenty-one. The rich, therefore, do not only live better, but longer than the poor.

But even the less opulent now possesses more means to restore his impaired health. It is not the least of the merits of medicine, that more efficacious and cheaper remedies are used now than at a former period. Before the Peruvian bark was discovered, how long had an artisan (exposed to the inclemency of the weather, and other noxious influences,) to suffer from intermittent fever and its consequences, and how quickly is he now restored to his occupation, more especially since the discovery of the alkaloids! Formerly, if an artisan had contracted a lameness of his arms by inhaling the vapour of lead, he remained for a long time a burthen to his family, and under the most favorable circumstances was obliged to consume, during his forced inaction, what he might have saved at a previous period. Now, by the aid of strychnine, and sulphur-baths, the surgeon speedily dismisses him, freed from pain and spasm, to his former occupation: wherefore it is that the present higher degree of medical skill in the use of simpler and surer modes of cure, the result of general instruction and an improved civilization, constitutes an important item among the causes of the decrease of maladies. Medical arrangements in general have acquired, almost everywhere, a reputable degree of development. For instance, natural and artificial baths are within the reach of every one who is in want of them. In consequence of improved communications, efficient drugs are now brought from all quarters of the globe at a moderate rate; others have been discovered by the exertions of our chemists. On this account, the number of incurable or painful chronic diseases is constantly more confined.

Through means of improved diagnostics, as well as of improved treatment both in medicine and surgery, more individuals of all ages are now preserved than at any former period. Even at the beginning of the present century the inflammations of the cerebral membrane, of the lungs, and of the intestinal canal of children, were for the most part mistaken, and therefore terminated unfavorably. The mode of cure of syphilis and its consequences was formerly as deleterious for the patient as the disease itself. Aneurism, which formerly was mostly fatal, is now successfully removed under the hands of skilful operators, by means of the ligature of large branches of arteries. Distortions of the limbs, which formerly—for instance, club-foot—impeded their use, are now removed by early and skilful operation. Squinting, which prevented many from improving their condition, is now as easily cured by a simple incision; so also is stammering, by systematic exercises in the mode of speaking. How many blind persons were once considered incurable, to whom sight is now restored in a moment!

It may not be urged as an argument against all we have hitherto stated, that new names of diseases are every day starting up,—proving, as is alleged, their increase. But names are not entities. As little as a botanist increases the botanical riches of a country by making new species and genera from mere varieties, as little does a nosologist, who transforms mere symptoms into forms of disease, prove the distinct existence of such forms. Fortunately, the number of diseases has not increased in reality, but only on paper; it is merely the modes of classification, and not the ailments themselves, which have been multiplied.

It often depends merely on the ideas received at college, or on certain fashionable notions of the faculty, whether or not some diseases are considered as epidemic and far spread. Thus, at one time we hear everywhere of affections of the heart, at another of inflammations of the abdomen, of the dorsal spine, &c.; whence it might appear as if heavy visitations were afflicting mankind in an extraordinary degree. Soon, however, this notion will be found illusory, and everything will return to the usual course of disease and recovery.

But even supposing that any particular disease be observed more frequently, where is the proof that it has really become more frequent? Among savage nations, a great many of the sickly offspring die in the first years of life; among civilized people, on the other hand, they are mostly preserved, and only at an advanced age fall a prey to natural or accidental death. Can it, then, be justly said that a disease has increased, merely because a greater number of persons perish by it from the circumstance of their attaining a higher age? Civilization has only the power of diminishing or preventing the tendencies to disease; it cannot confer on man physical immortality! Considering the greater amount of vital powers and vital excitement among civilized people, the victory over the many dangers which threaten them, is the more glorious.

As a proof of the justice of the foregoing statements, we might adduce the historical or statistical data of almost every disease. We select, in the first instance, some of the principal.

*Consumption.* It has often been asserted, that pulmonary phthisis—the great destroyer of the human kind, and that, too, in the very prime of life, when mind and body are full grown and developed—has increased in these times, compared with former times: but this position we cannot admit. Comparative tables, which alone can lead to the solution of such a question, do not yet exist, even in a form which may be deemed approximatively correct, and cannot, therefore, be depended upon. One of the most recent authors, (Sir James Clark,) who has given a statement of those who have died in London from that disease, from the year 1700 to 1821, says: "The opinion entertained by some authors, that phthisis has increased since the year 1750, is based on the error, that its relative fatality has been placed in comparison with that of all other diseases, instead of comparing its absolute fatality with the general amount of population. That relative increase is not occasioned by the increase of phthisis, but by the decrease of other morbid affections: the causes which have had such a beneficial influence on the mortality arising from other diseases, have passed away without producing any influence on phthisis." Also, if we compare the statistical tables for a number of years, compiled in countries where they have been made out with care, (for instance, in Würtemburg,) we shall find, not an increase, but a decrease of this disease. Since the year 1787 a smaller number have died annually of phthisis at Stuttgart. During five years only *three* individuals have been exempted on that account from military service. In some districts there was not one person affected with this disease.

The ancients speak often of phthisis, and mention its hereditary and even contagious characters. But just as we do not confine the term consumption to the pulmonary affection, they also used the word phthisis in a wider acceptation. Some countries are little favorable for the development of this disease, owing to their position and endemic circumstances; for instance, Egypt: and it was stated, even very recently, that the disease scarcely occurs there at all. It has also been stated, that in countries where aqueous exhalations caused intermittent fevers, phthisis is little known.

Admitting that, under similar external circumstances, phthisis makes its appearance equally among civilized and savage nations, the mortality among the latter will doubtless be much greater; as we know that, without a very careful regimen of life and appropriate professional aid, it will be soon accompanied by inflammation, and then degenerate into florid consumption, or will be soon followed by a softening of the pulmonary tissue, and all the symptoms of tubercular decline.

Since the deleterious influence of such trades as affect the lungs—as, for instance, that of grinders, brushmakers, &c.—and since the evil effects of mercurial vapours, and the consequences of the too great use of mercurial ointment have been properly understood, and means taken against them, phthisis has been often prevented. And as medical investigation has been so strongly directed to the nature and cause of the deposition of tuberculous matter in the lungs, we may still entertain a hope, that that morbid almost inorganic mass may be checked in its growth, and be made capable of absorption and excretion.

In conclusion, we ought to observe, that the deductions derived from tables of mortality (although they are at present the only data to go by,) are to be accepted with caution; because, when we find it stated that a patient died of pectoral disease, whether chronic catarrh, a spitting of blood, &c., it remains still doubtful whether the disease was phthisis or not.

*Scrofula.* This malady, which especially affects young persons, may certainly be considered rather on the decrease,than otherwise, as, by the present improved circumstances relating to the dwellings of man, the elements of its formation become less numerous. By the greater attention paid to the cleanliness of the skin, and to the mucous membrane of the intestines, which is much facilitated by the greater cheapness of digestible food, the disease does not manifest itself so easily, or if it does so, it is checked before it has attained any degree of development, by abstaining from food containing gluten, (Hänkmehl,) by exercise in the open air, by dwelling in a pure atmosphere, avoiding of damp habitations, and by the use of cool baths.

The old physicians have described this disease very well, but their way of treating it was not always the most judicious: they considered it a mere external one, and endeavoured to bring the swellings to suppuration. Out of all the numerous anti-scrofulous modes of cure and eulogised remedies presumed specific, recourse has finally been had to diet—certainly a great improvement.

*Rickets.* This disease, formerly described by the Arabians under the name of hunchback, and arising from fever, as observed, during the sixteenth century, in

Holland and Switzerland, and which was so well described from observations made in England in the seventeenth century, that it received in Germany the name of "English disease," becomes less prevalent every year, from the same causes as have been stated under the head of Scrofula. The so-called *double limbs* (doppelten Glieder) and other distortions, now appear in less number; and if they are more talked of, and even especial institutions established for their cure, it is to be recollected, that the increased number of journals calls forth many detailed descriptions of subjects which formerly were passed over in silence. Besides, the results of former methods are now regarded as insufficient: it is not only health we now seek for; beauty and strength are desiderated in addition.

*Syphilis.* Among the most awful diseases which have long afflicted mankind, and still continue to do so to a certain degree, syphilis occupies a prominent place. I shall not enter here upon the question, whether it was known to antiquity, and whether it also occurs in animals; suffice it to say, that when it made its first appearance, some centuries back, it soon spread with great rapidity, and was followed by a number of accessory maladies, unlike any other ailment to which flesh is heir: since then, its baneful sway has successively extended over the whole inhabited globe. At its first advent, the guilty, as well as the innocent, were seized by it: the harmless babe—nay, the unborn embryo—was not secure from its poisoning influences. All the remedies which the then rude and dark ages taxed for checking it, proved useless, or even increased the evil; and thus it seemed, that this ominous ailment—affecting as it did the very sources of propagation and increase—even menaced the existence of the whole race. The disease has not been extirpated: it still exists, but civilization and science have much curtailed its extent, its intensity, and its consequences. In the same ratio as the knowledge of its essential relations to the organism and the remedies employed against it have increased, so also has the morality of states and nations improved; and it would be easy to prove how both these causes have contributed towards the checking of its progress, as well as mitigating its symptoms and complications; and how it has finally become restricted within rather narrow limits. It cannot be denied, that that very remedy which was considered, for a long time, indispensable and unavoidable, was not less detrimental to health than the disease itself. But we now know, full well, that the untoward symptoms alluded to were not so much to be attributed to the use as to the abuse of this remedy; so much so, that the conviction of our being able entirely to dispense with it is becoming every day more general. As syphilis is much milder in warm climates, (for instance, in Egypt,) so much so as to be curable by nature, it was imagined that energetic therapeutic measures were required in our northern latitudes. But in proportion as it is acknowledged that the affections of the cutis and the mucous membranes comprehend the chief therapeutic indications, and that a radical cure can be effected without the use of mercury, the character of the disease has become more simple, and the organism less subject to secondary ailments.

The better knowledge of syphilitic affections, together with that of the disease called Marsch-krankheit in Holstein, the radesyge and spetälska in Scandinavia, the sibbens in Scotland, the skerlievo on the shores of the Adriatic, the Krimmean disease (krimmschen-krankheit) on those of the Black Sea, the Asturian rose, the eruptive disease of Aleppo, the yaws and pians, makes us often believe in an affinity between syphilis and lepra, or, at least, doubt of the pure specific character of the former. This disease, therefore, formerly so terrible and mysterious, has now come within the range of ordinary maladies, being not only amenable to art, but vincible by art in every respect. It is with some degree of confidence that the philanthropist may expect its gradual decrease and disappearance, at a period not far off; if those authorities to whom devolve the superintendence and promotion of the general health, as well as the maintaining of public morals, will not relax in their exertions.

In like manner as the above three malignant and most extended plagues of mankind are not only not ascribable to the progress of civilization, but are, on the contrary, combated by it with all possible might, so also is the case with almost all other ailments; inasmuch as the representatives of civilization (we mean science, art, and morals,) ever oppose them, either in an open or indirect manner. For the sake of placing this matter in a clear light, it will suffice shortly to illustrate some more prominent forms from the different departments of pathology.

*Nervous diseases.* The civilization of the ancients had attained such a high pitch, that we view the remnants of their architecture and sculpture—we regard their poets and historians with astonishment and admiration, as the monuments of some finer world, now passed away! The economy of their public and private life comprehended such a chequered tissue of manifold arts and enjoyments, that the reproaches made against our own age, stigmatizing it as one of too much refinement, are perhaps unfounded. But even supposing that modern civilization does not possess the simple grandeur of ancient thought, and has merely adopted the superfluous wants, the appetites and passions of the olden time, or only combined these with the views and notions of a newer order of things, still it is striking that, in the department of *nervous diseases*, no new forms of these anomalies of over-excitation are to be met with. Nay, the circle of nervous diseases has not only not become wider, compared with former times, but has been rather restricted. This will appear evident when we compare the writings and traditions of antiquity with the records of the present time.

*Nostalgia.* Even within the recollection of practitioners still living—a period which, compared with history, is obviously of no account—a dangerous nervous disease, the *nostalgia*, has nearly ceased. It is well known how much this formerly prevailed, especially among the natives of mountainous countries; and how very seldom is it now even heard of! Into the heretofore lonesome valleys of the Highlands a more lively traffic has found its way; the inhabitants have entered into communion with the whole world; and the accelerated communication, by means of stage-coaches, steam-vessels, and railroads, has taken away from the stranger in a foreign land the depressing idea of secluded loneliness and cold isolation; letters from one's home now arrive with astonishing celerity, as if by pigeon-post; the mere idea that the decision of a moment may realize the possibility of being transported, as on the wings of the wind, to the place of our anxious desires, banishes every feeling of hopeless despair.

*Hysteria and hypochondriasis.* The easy modes of travelling now prevalent, and the constant custom of availing ourselves thereof, are among the chief reasons that hypochondriasis and hysteria are now less frequent than formerly. Travelling is one of the chief preventives of these maladies; not merely from the necessary bodily exercise connected with it, but from the enforced change of the mode of life, from the necessity of entering into foreign feelings, and the unavoidable impulse of new and distracting impressions. In proportion, also, as with less trouble and expense than heretofore, that abode can be chosen which is most suitable both to mind and body, the duration of life is also increased. The increase of strength which the whole constitution obtains, at times, by a change of climate often surpasses belief. The deeper organic anomalies existing in hypochondria and hysteria can also be now the easier remedied, as the organs which are thereby affected—especially those which are connected with the sympathetic nerve—are much better understood than they were formerly; and, consequently, better remedies can be adopted against them. It is proper, moreover, to observe, that whoever should conclude from the infrequency of the terms hysteria and hypochondriasis among ancient authors, that the diseases themselves were proportionally rare, would commit a great error; as, according to the theories of those times, several other appellations have been applied to these diseases; for instance, *dry cholera, the spleen*, &c.

*Chorea Sti. Viti.* This is one of those nervous diseases which certainly was known formerly, but was comprised with others under other appellations. As it mostly appears in the so-called period of development, its decrease is to be expected with the more certainty as medicine is constantly studying the changes of the human body, and education becoming every day more circumspect. Indeed, cases of the disease are now rather rare.

*The dancing mania.* This disease was a consequence of the ties of public and private life having been so much loosened, in consequence of the ravages of the *black death.* Many persons were then seized, as if by some contagious plague, with a demoniacal furor to stroll in loose bands through the land, and to dance until they suffered complete exhaustion, which was followed by feigned or real spasmodic affection. With the exception of those who were carried away by the impulse of imitation, the subjects of this delusion were mostly persons who had long lived on public charity; and they persisted until authority had regained its lost influence, and social life assumed a degree of reasonable quiet. In a well-regulated state of society, like the present, the repetition of such an epidemic is scarcely possible. It is true that our present time has exhibited something similar in the *preaching mania* of Småland, in Sweden; but this strange excitement was soon assuaged by the cooperation of all sensible persons.

*Catalepsy* (*Starrsucht*). This disease has become rather a subject of history than of nosology, since the doubt of its existence as a real disease has grown into conviction.

*Raphania* (Kriebel-krankheit), which, on account of its striking symptoms, has been named also "spasmodic tragedy," is only to be met with in districts where no measures are taken against the growth of ergot in corn. The simplest remedy is to dry the land by draining. In some countries the government issues public notifications how to guard against the bad effects of the corn, which might have been spoiled in wet seasons by this poisonous fungus.

*Paralysis.* Palsy, more especially of the lower extremities, is a disease so frequently observed in modern times, that it cannot be denied, that it has numerically increased. As it is, however, very probable that the recent great political revolutions, with their concomitant excitement and disasters which befel so many persons, as well as the hardships of war, have a great share in its increase, we may presume, that the disease will again decrease in frequency during a lasting peace and a consolidated social condition. This, certainly, will be much assisted by the recent discoveries as to the function of the dorsal nerves, as well as the knowledge of the effect of some vegetable alkaloids.

*Neuralgia.* Nervous pains in general, and especially tic douloureux and angina pectoris, have been much noticed since the beginning of this century. Still it is difficult to say anything positive about the alleged increase of this disease, as its existence previously is not properly elucidated. The Arabian physicians speak often of painful spasms of the face; and one of them even recommended to apply derivatives near the origin of the nerves. Several of the great men of antiquity are said to have suffered from the angina pectoris.

*Hydrophobia.* This disease is now not known in many countries but by hearsay, whilst in others it still claims its share of victims. As art is here without avail when the disease has become somewhat developed, every care is to be taken to prevent it. The modes of doing this are various, and have proved efficacious in so many instances, that we may cherish the hope, that if generally resorted to and thoroughly carried out, they will finally eradicate the evil. Considering the fact, that house-dogs are those which usually run mad, several governments have imposed a tax, with the view of diminishing their number. At Argos a feast was celebrated in ancient times, in the dog-days, called *kynaphontis,* at which a number of dogs were publicly killed. And it is to this expedient that we shall be obliged to resort if we do not like to be taxed, or if taxation does not prevent the keeping of too many useless dogs.

*Delirium tremens.* It is probable that this affection has only become a distinct form of disease since the mode of distilling ardent spirits has been so much improved, that large quantities can be had at a moderate price—a circumstance which so much encourages intemperate habits. This, however, has reached its climax. The general disgust, temperance associations, as well as the improved therapeutic indication, to remove the morbid state of the patient by inducing sleep, are all directed towards the same aim.

*The gilders' malady* (Zittern der Vergolder), which renders invalid so many persons obliged to inhale mercurial vapours, and which often ends in complete paralysis, has lost much of its awful character by the knowledge of the salubrious effects of chalybeates, taken either internally or applied in the shape of baths. The newly-invented method, by which almost all metals can be gilt by galvanic process in a substantial and solid manner, will probably lead to a perfect abandoning of the pernicious quicksilver.

*Colica pictorum.* This disease is also a marked proof of the present progress of medicine. Persons affected with this disease are now generally cured by an appropriate alterative method. It was chiefly owing to the practice of the Romans, to inspissate the juice of grapes in leaden vessels, and to preserve their wine by the admixture of this syrup, that they were so much subject to colic. And it is on account of lead being now more cautiously used for the domestic purposes of glazing and tinning vessels, as well as in the fabrication of oxide of lead, and even for medicinal purposes, that we are to attribute the diminution of the causes and, consequently, of the effects of poisoning by lead. And the same happy result would be seen in the case of painters, if they would resolve to use the oxide of zinc instead of that of lead.

*Congestions.* As often as the unavoidable evils which extended and refined civilization has brought on are spoken of, the congestive diseases are always mentioned; for instance, the disordinate tendency of blood towards the head, chest, and abdomen, caused by unnatural positions, peculiar movements of the body in working in secluded rooms, also by improper garments and too stimulant food or medicine. It is more especially the hemorrhoids which are counted amongst these artificial diseases. Although it may be the case that the mucous membranes and the vessels of the digestive organs are more affected with us, on account of our social enjoyments, than they are in a natural state; still, even in the latter state, the disease is to be met with, as a self-aid of nature, which thus endeavours to produce a derivation from more noble organs. They terminate seldom fatally, and are mostly the consequence of an inappropriate and irregular mode of life, coupled with the neglect to remove the disposition and to repress the early growth of symptoms. The hemorrhoids have not what may be called any morbid seed in the human body which must necessarily develop itself; it lies mostly in the free will and conduct of the individual, whether he shall be thus affected or not.

*Inflammation.* Inflammatory irritations and real inflammations have mostly on that account increased, as well numerically as in intensity, because civilization has extended from the milder Asiatic and South-European climate to more northern latitudes. So soon as man, with enlarged social and physical wants, comes in contact with a more severe climate, it follows of course that he must experience the full influence of its inimical agencies. Where change of temperature takes place suddenly, and sharp gales frequently displace more genial breezes; where a humid atmosphere and thick and heavy fogs prevent the access of the milder rays of the sun—more occasion will be afforded for the formation of rheumatism, measles, and catarrh. But even in this case it belongs to a state of civilization, with its many resources,—such as appropriate clothing, warm dwellings, due exercise, increased strength of constitution, and an invigorating mental activity,—to wage successful war with the influences of the climate, and even to achieve more than the rude son of nature, merged in his stupid inactivity, can do, viz., just to manage to subsist on the extremest limits of the habitable earth.

*Ophthalmia.* The Egyptian inflammation of the eyes, which has become so prevalent in the present century, is nothing else but an intensive catarrhal inflammation, the discharged phlegm being of a corroding and, if the disease has reached its height, of a contagious character. The ancients were acquainted with it, and its spreading is not to be apprehended, if its contagious character is properly taken notice of.

*Croup.* This disease, which only a few years past, was the terror of all parents, when viewed in reference to its peculiar intensity and frequency, may be regarded as a produce of recent times—a sad result of our social condition and the physical education of our youth. The employment of emetics early in the disease has proved how it can be checked with perfect success; and, consequently, it can no longer be considered as one of the ominous satellites of our times, whatever may be its origin or nature.

The men of civilized nations, as they feed better, are more subject to pure inflammation than those of uncivilized, because they are more plethoric and excitable, and, in the main, undergo greater toils and perils.

*Fever.* Fevers, both inflammatory and nervous, have lost much of their dangerous and fatal characters, since we have advanced more in our knowledge of them, and since their prevention has been more attended to; those already existing have been treated with more skill, and those which threatened to be contagious have been skilfully prevented. The worst forms of fever are not everywhere the same; some countries are entirely free from them, whilst others are ravaged by them most severely. In the latter case the merit of checking them is so much the greater. It is said that Egypt and India can neither generate nor propagate typhus. How different is the case with Ireland! But it is in this last instance shown most clearly what marvels the exertions of medical men can accomplish when they are assisted by the authorities, and even by the people themselves. It is difficult to ascertain why certain periods and countries remain free from this disease, while others are severely suffering under its affliction. Civilization certainly has no share in its production, but strenuously assists in checking it. The ancients speak only of malignant nervous fevers. The slow nervous fever was well known to the Arabs, who treated it with cooling medicines. Afterwards, this form of disease was but little noticed; and even medical men of our times assert that it did not make its appearance before the close of the last century. The petechial typhus, accompanied by typhomania, has much decreased, in comparison with former times. The English sweating-fever, which did much mischief in the fifteenth and sixteenth centuries, has long ago ceased. The Hungarian fever, which made its appearance in the shape of a nervous putrid fever, and in the camps, and which was accompanied by the most violent colics, and spread contagiously, is now only known historically.

*The plague.* The Oriental plague, which, under the name of *black death,* spread, in the fourteenth century, over the inhabited globe, like some winged demon, and made those horrid ravages in Vienna and London in the middle of the seventeenth century, and destroyed in the eighteenth nearly one third of the inhabitants of the country of Brandenburg, and in Dantzig nearly one half, and which, even in our days, is constantly penetrating into Europe wherever isolating measures are not properly kept up,—is one of the most striking examples of the victory of civilization over barbarism. Whilst Mahomedan nations look on indifferently at the uninterrupted spreading of this plague amongst them—its destroying fire, scarcely put under, anon reviving from its ashes—whilst they consider it as an unavoidable destiny, when their nearest relatives die away in rapid succession—whilst they, in the height of their carelessness, retain the objects used by their late friends, and treat the malady with imaginary remedies, such as mummies, bezoar, and amulets—whilst all this is done among the Turks, the Christian nations, on the other hand, seek, by isolation, cleanliness, a strict diet, and a scientific treatment, to guard against it; to establish quarantines, which combine the greatest attention to health with the comfort of the traveller, as far as it can be attended to safely; so much so, that a most lively traffic and communication, as well by land as sea, is entertained, without that one single life is endangered. It is even owing to civilization, with its unremitting advice and interference, that in the very seat of the plague the tenets of fatalism are no more attended to, but that the natives listen to the precepts of a sound medical policy. The hope of philanthropists, that the time when even those barbarous nations will enjoy the blessings of civilization, by being freed from the plague, is not distant from its accomplishment.

*The yellow fever.* The yellow fever has even visited of late the shores of the European continent, and left behind the salutary admonition, that only great care and rigorous isolation will prevent its return. If the observation should be confirmed, that the germ of this disease is mostly generated on board slave-ships, by the effluvia of the Negroes crowded together, the general prohibition of the slave-trade ought to be the more urged, as an affair of humanity and of science, on which medicine ought to insist on its own account.

*Ague.* Intermittent fevers (of which it may be said, that they are more than any other disease, rooted in the soil,) are gradually losing ground and disappearing. If the attempts to regain extensive marshes for cultivation and traffic are persevered in—if the draining of stagnant waters, and the prevention of inundations in the neighbourhood of the sea, &c. are duly pursued—if, in districts where rice or hemp is cultivated, the deterioration of the air is prevented by the planting of trees—if the cleansing of sewers and the filling up of moats are attended to—if this care is extended to the procuring of pure water for drinking, good food, and cleanly dwellings—marsh-fevers will cease, even although they had obtained the right of denizenship, and seemed to have become endemic. The history of the economical improvements on the surface of the soil affords the best proofs of the truth of our assertion. The number of diseases and deaths gradually decreases; and those once dreaded localities—as if the abode of some fabled dragon—are even sought after on account of their salubrity.

*Dysentery.* This disease, which was formerly so severe an affliction, and spread over large tracts of country, has progressively undergone a remarkable decrease, as well in the numbers of the subjects affected, as in the intensity of the affection. There scarcely remains a doubt that this improvement does not so much depend on epidemic influences, as on the promulgation of better dietetic rules, the production of more wholesome food, and the more general regard now paid to adequate nourishment, clothing, residence, warming, &c.; and, in the case of the disease being threatened, to the adoption of more effectual preventive measure.

*Cholera.* The Asiatic cholera—the strange produce of climatorial and national causes in the East Indies—has lately fallen, like a new disease, on Europe. But certainly its sudden invasion and gradual disappearance alike demonstrated the existence and the benefits of a higher state of civilization. It was only under the shelter of transient political and warlike turmoils, that it was ever possible for the disease to transgress certain limits, which, otherwise, it could have never done. The means resorted to by one of the German governments to protect its territory from the approaching plague will remain memorable in the history of its European migration. The resolution to combat the approach of the invading enemy, step by step, was the result of the examination of extensive data, officially investigated. Here, as in every case where we do not yield to illusion, we succeeded, in numberless cases, by a rigid, complete, and consequential carrying out of the sanitary regulations, and especially by the isolation of the infected, and the careful cleansing of the dwellings, to suppress the evil in its very germ, and to check its further spreading. At the period of this danger there were also manifested such an amount of sympathetic feelings, such care for the support of the poor and suffering, such attention of the authorities to the cleansing of dwellings, as well as such an assiduous cooperation of all medical men towards effecting everything which human art and experience afforded towards the eradication of the disease, that we at length succeeded to vanquish it step by step, and finally to expel it utterly from Europe. As, in this case, root and seed seem to be destroyed, it is to be hoped that the acquired knowledge of its true character will prevent its again returning to us from its far-distant original abode.

*The chimney-sweeper's cancer,* which existed especially in England, has been expunged, as it were by act of parliament, from the series of pathological phenomena. The mode of cleansing of very narrow chimneys by boys had occasioned this disease; and the new regulation, to clean such by the machines only, removed both cause and effect.

*Scurvy.* The sea-scurvy is one of the incontrovertible proofs that advancing civilization is capable, by the searching out of the right means, and their careful application, to take away from even the severest ailments their strength, nay, to destroy their very existence. The orders of the English Admiralty, to supply all vessels, which undertake long voyages, with a sufficient quantity of lemon-juice, made it possible for them to approach even the South and North Poles without a single case of this once so dreaded malady. In the government vessels, where the prescribed cleanliness and good food are strictly attended to, there has, of late, no case of scurvy been heard of; not so, however, in the vessels of private individuals, who neglect, on account of a sordid economy, to keep a medical man on board, and to provide the necessary store of lemon-juice and fresh provisions. It is in vain that we now look in the naval hospitals for this form of cachexy: whoever would now know anything of it must look back into books, or in situations where the precepts of civilization are not attended to.

*Lepra.* This was the most dreadful among the popular maladies of antiquity, and attacked, under different forms, even the higher classes: with us it has almost vanished, leaving scarce a trace behind. In former times, thousands of leper-houses served for the reception of those who suffered from the most severe forms of this most contagious disease; now it is a rare occurrence, whenever a slight modification of it makes its appearance anywhere. It was principally by means of the knights returning from the East during the Crusades, that this disease was spread in Europe; but the development of our civilization has driven it once more back to the countries where the low state of medical art, the indolence of the people, superstition, and ignorance may yet probably for a long time afford an appropriate shelter.

*Smallpox.* It is yet doubtful whether the smallpox was known to the ancients; it is too certain, however, that this disease was considered for many centuries as one of the unavoidable calamities of mankind: it has nearly disappeared since the discovery and introduction of the vaccine. Of all the blessings which have ever been bestowed by man on man, none has proved greater and more important than this. Up to the epoch of its discovery, about a twelfth part of the population of the globe had been either destroyed, or deprived of their health, or disfigured by this plague. To such ravages this inestimable process put a sudden stop, as if by the wand of a magician. The occasional appearance of the real or modified smallpox since then, cannot be placed in comparison with what took place before. Even at the time when it was believed that a single and incomplete inoculation was a sufficient guarantee, and every other precaution was neglected, the mortality, compared with former times, had extremely decreased. And now, in all countries where vaccination has been placed under official control, where care is taken that a sufficient supply of efficient virus is provided, and where revaccination is duly attended to, the smallpox has ceased to exist as a mortal disease. Vaccination is a splendid proof of the advantages of civilization, the most precious result of human thought and research, the fairest reward for devoted attention paid to the delicate and, at times, almost incomprehensible hints and indications of nature. The health and life of millions are preserved through its agency, without any sacrifice of pain or privation. This discovery, like the armed Minerva from the head of Jove, sprung at once, perfect and efficient, from the studies and experiments of its great discoverer. Right speedily it accomplished its triumphal march through the world, reaching even the most distant Indians, from whom there is still a letter in existence, addressed to the discoverer, in which they thank him for that gift of the Great Spirit. Thus it results, that the undeniable blessings of civilization reach even the countries of the remotest savage, and make him more ready to receive the other advantages of civilized life, which may be less obvious to his rude perceptions.

To conclude: Do we need any more enumeration of diseases, in support of the fact, that civilization not only does not increase them, but diminishes and partially eliminates them? Almost every one of the innumerable ailments to which flesh is heir, affords, when thoroughly investigated in its causes and relations, a new proof of this consoling truth. In the same proportion as arts, sciences, morals, and refinement advance, so also are the means multiplied whereby human life is strengthened and protected, as well against internal as external foes. True knowledge and true welfare march hand in hand together. As in the Grecian

Mythos of old, light and healing are the same. Phœbus Apollo who illuminated the world, was also its helper; to him was sung pæans, the hymns of salvation. What previous ages clothed in mythical garb, has become truth in the lapse of centuries. The nearer man arrives at the full consciousness and development of his powers, the more surely will he also attain the full harmony of corporeal existence. It can be therefore asserted, with perfect consistency, that knowledge is not only power, but even health. The approach to knowledge is now forbidden to none; the printing-press and the schools afford to every one participation in the the highest goods of mankind. And MEDICINE has not kept behind the other promoters of humanity. As its glorious aim has always been to eradicate diseases or to abate their violence, to assist the suffering, to strengthen the healthy, so has it also endeavoured more and more to make its truths the common patrimony of mankind, and the undeniable evidence of civilization.

---

### REPORT ON THE CHANGES IN THE BLOOD IN INFLAMMATION, AND ON THE NATURE OF THE HEALING PROCESS.

By T. Wharton Jones, F.R.S.

**Lecturer on Anatomy, Physiology, and Pathology, at the Charing-Cross Hospital; Corresponding Member of the Imperial and Royal Society of Physicians and Surgeons of Vienna, &c.**

